# A278 SEX DIFFERENCES IN THE EFFECT OF THE MICROBIOTA FROM IRRITABLE BOWEL SYNDROME PATIENTS ON ABDOMINAL PAIN

**DOI:** 10.1093/jcag/gwac036.278

**Published:** 2023-03-07

**Authors:** A Bennett, C Baker, M Guzman-Rodriguez, N Jimenez-Vargas, S Vanner, D Reed, A Lomax

**Affiliations:** 1 Department of Biomedical and Molecular Sciences; 2 Department of Medicine, Queen's University, Kingston, Canada

## Abstract

**Background:**

Irritable bowel syndrome (IBS) is a chronic abdominal pain disorder that affects women twice as often as men. The gut microbiota has been implicated as a key player in the modulation of abdominal pain in IBS. Given this, we hypothesised that the production of pro-nociceptive mediators within the gut lumen are increased in females, and this contributes to the female predominance of IBS.

**Purpose:**

Compare the effects of FS from male and female IBS patients on abdominal pain pathways and identify the impact of female mouse estrous cycle on abdominal pain.

**Method:**

Fecal supernatants (FS) were perfused through murine colonic preparations while performing extracellular colonic afferent nerve recordings to measure changes in action potential frequency in response to colonic distension. Phase of estrous cycle in female mice was determined through vaginal swabs. FS from male and female IBS patients reporting low, moderate, and high levels of abdominal pain were used.

**Result(s):**

FS from female IBS patients (N=6) increased afferent nerve discharge (p < 0.05) whereas FS from male IBS patients has no effect (N=4). However, single unit analysis of nociceptive axons revealed that male IBS FS increased nociceptor activity in female mice taken during the proestrus/estrus stage (p < 0.05), but not female mice taken during the metestrus/diestrus stage or male mice. Further investigation found that IBS FS from female patients with high abdominal pain (N=6), but not patients with moderate (N=5) or low pain (N=3), increased visceral afferent nerve discharge by 70%. Single unit analysis of nociceptive axons showed that their activation was increased by almost 50% following FS perfusion from high abdominal pain patients only (p < 0.05). Histamine concentrations and proteolytic activity are increased in FS from female IBS patients with high abdominal pain compared to male IBS patients.

**Conclusion(s):**

This work suggests that luminal mediators that impact abdominal pain are increased in female IBS patients compared to male IBS patients, and females appear to be more sensitive to their pro-nociceptive effects. Together, these sex differences may contribute to the female predominance of IBS.

**Disclosure of Interest:**

None Declared

